# Hospitalisations for respiratory syncytial virus bronchiolitis in Akershus, Norway, 1993–2000: a population-based retrospective study

**DOI:** 10.1186/1471-2431-4-25

**Published:** 2004-12-17

**Authors:** Hans-Olav Fjaerli, Teresa Farstad, Dag Bratlid

**Affiliations:** 1 University of Oslo, Faculty Division Akershus University Hospital, 1474 Nordbyhagen, Norway; 2 Department of Paediatrics, Akershus University Hospital, 1474 Nordbyhagen, Norway; 3 Department of Laboratory Medicine, Children's and Women's Health, Norwegian University of Science and Technology; Department of Paediatrics, St. Olav University Hospital, Trondheim, Norway

## Abstract

**Background:**

RSV is recognized as the most important cause of serious lower respiratory tract illness in infants and young children worldwide leading to hospitalisation in a great number of cases, especially in certain high-risk groups. The aims of the present study were to identify risk groups, outcome and incidences of hospitalisation for RSV bronchiolitis in Norwegian children under two years of age and to compare the results with other studies.

**Methods:**

We performed a population-based retrospective survey for the period 1993–2000 in children under two years of age hospitalised for RSV bronchiolitis.

**Results:**

822 admissions from 764 patients were identified, 93% had one hospitalisation, while 7% had two or more hospitalisations. Mean annual hospitalisation incidences were 21.7 per 1.000 children under one year of age, 6.8 per 1.000 children at 1–2 years of age and 14.1 per 1.000 children under two years of age. 77 children (85 admissions) belonged to one or more high-risk groups such as preterm birth, trisomy 21 and congenital heart disease. For preterm children under one year of age, at 1–2 years of age and under two years of age hospitalisation incidences per 1.000 children were 23.5, 8.7 and 16.2 respectively. The incidence for children under two years of age with trisomy 21 was 153.8 per 1.000 children.

**Conclusion:**

While the overall hospitalisation incidences and outcome of RSV bronchiolitis were in agreement with other studies, hospitalisation incidences for preterm children were lower than in many other studies. Age on admission for preterm children, when corrected for prematurity, was comparable to low-risk children. Length of hospitalisation and morbidity was high in both preterm children, children with a congenital heart disease and in children with trisomy 21, the last group being at particular high risk for severe disease.

## Background

Respiratory tract infections due to respiratory syncytial virus (RSV) are very common in young children worldwide. In temperate climates the infection occurs in yearly winter epidemics, and by two years of age most children have been infected [1]. Reinfections are common throughout life but the first infection is usually the most severe [2]. Symptoms vary from a mild upper respiratory tract infection to a severe bronchiolitis with hyperinflated lungs and hypoxemia [3]. Children in the first months of life, particularly those with preterm birth, underlying chronic lung disease (CLD), congenital heart disease (CHD), neuromuscular disease, airway malformations or impaired cellular immunity are at risk for severe disease [4-6].

Local epidemiological studies are important when considering new strategies for the prevention of RSV bronchiolitis. An example of such a strategy is the monthly injection of the humanised murine monoclonal antibody palivizumab to certain high-risk groups, which in many studies has shown a significant reduction in the incidence of hospitalisation for RSV bronchiolitis [7].

The aims of the present study are to identify risk groups, outcome and incidences of hospitalisation for RSV bronchiolitis in Norwegian children under two years of age, and to compare these results with other studies.

## Methods

### Background population

Akershus is a large suburban and countryside region located around the capital of Oslo with approximately 10% of the total population in Norway. The Paediatric Department at Akershus University Hospital (Ahus) is the only hospital for children under 15 years of age living in the 20 northern, southern and eastern communities of the region and serves all kinds of hospital services, except for paediatric surgery. Deliveries have been rather stable at approximately 3.500 per year in the service area during the last decade. Due to a local agreement infants from approximately 500 unselected term deliveries from our service area are born at Rikshospitalet in Oslo each year, but these infants are admitted to our hospital in case of disease during childhood. The number of children under two years of age living in the service area at any time during the follow-up period were identified from official Norwegian Population Statistics . The number of children under two years of age born before completed 37 weeks of gestation in the service area during the study period and still alive after the neonatal period was identified from the Norwegian Medical Birth Registry . All children with trisomy 21 have a program for follow-up from birth in our hospital and the number of children under two years of age with trisomy 21 was therefore identified from in-patient and out-patient hospital records. The number of children in the general population with a CHD was not known in the present study and hospitalisation incidences could therefore not be estimated. Overall mortality after the neonatal period as well as the number of children moving out of the service area during the study period were low and have not been corrected for.

### Study population

The present study is a population-based retrospective survey on children under two years of age admitted to the hospital with a diagnosis of bronchiolitis (ICD 9 & 10) during the period February 5^th ^1993 to January 31^th ^2000 and diagnosed as positive with RSV in nasopharyngeal aspirate (NPA). RSV was diagnosed by an enzyme-linked immunosorbent assay (ELISA) for RSV (Abbott TestPack RSV). The test is simple and easy to perform giving a result within 30 minutes. The sensitivity, specificity, positive and negative predictive value is stated to be 74–92%, 86–100%, 81% and 93% respectively [8,9].

764 children who fulfilled the diagnostic criteria were identified. A total of 77 children belonged to one or more well known high-risk groups. Of these, 58 children had been born before completed 37 weeks of gestation, some with additional diseases such as CLD (12 children) and CHD (four children). 12 children had a diagnosis of CHD as the only additional disease and seven children had trisomy 21, four of these also diagnosed with CHD. These 77 children were regarded as a high-risk group for subgroup analysis. No children with other well-known risk-factors (neuromuscular disease, airway malformations, impaired cellular immunity or others) were identified. The remaining 687 children were born at term and healthy and regarded as a low-risk group for subgroup analysis.

### Statistics

Data were analysed with the statistical programme SPSS, version 11. For analysis of gender we used the Chi square test and for comparisons between low-risk and high-risk groups we used the independent sample T-test. A p-value <0.05 was used as limit for statistical significance.

## Results

### Epidemiology

**Figure 1:**
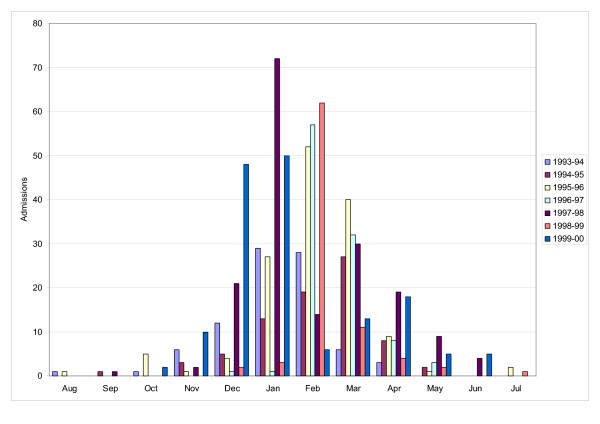
Seasonal variations by month and year in hospitalisations for RSV bronchiolitis in children under two years of age in Akershus, Norway, February 1993 to January 2000

During this seven years follow-up period 764 children (822 admissions) were hospitalised for RSV bronchiolitis. 93% (707 children) had only one hospitalisation and 7% (57 children) had two or more hospitalisations for RSV bronchiolitis during the first two years of life. The highest number of admissions was recorded during the winter months December-April, but cases were also found in late spring and early autumn. Both the number of admissions and peak-time varied between seasons but with no specific pattern of variability (figure 1). A significant male predominance was observed, with 517 (63%) of all admissions being boys (P < 0.001)

The majority of children (75% of all admissions) were hospitalised within the first year of life with children less than six months old being responsible for 45% of all admissions. Median age at hospitalisation was 6 months (range 0–23 months) and a median length of stay of 4 days (range 1–41 days) was observed (table 1).

**Table 1:** Age on admission and length of stay in children under two years of age hospitalised for RSV bronchiolitis in Akershus, Norway, February 1993 through January 2000

Risk groups	Children (no.)	Adm (no.)	Median age^a^	P-value (age)	Median stay^b^	P-value (stay)
All children	764	822	6.0		4.0	
Premature^c^	58	64	8.0	NS^e^	8.0	<0.001^e^
Corrected age^d^	58	64	5.4	NS^e^		
Trisomy 21	7	8	9.0	NS^e^	7.5	<0.001^e^
CHD*	12	13	7.0	NS^e^	6.0	NS^e^
All low-risk	687	737	6.0		4.0	
All high-risk	77	85	8.0	0.045^e^	8.0	<0.001^e^
Corrected age^d^	77	85	5.5	NS^e^		

^a^Age in months

^b^Days including admission day

^c^Born at <37 weeks of gestational age

^d^Corrected for weeks of prematurity

^e^Versus low-risk children

During the study period nine children (1.2%) developed severe respiratory distress and needed mechanical ventilation and two of them (0.3%) died. Four of these children had no known risk-factors for severe disease. However, the two children who died were both considered as high-risk patients, one of them was diagnosed with CHD and the other with trisomy 21 as well as CHD.

Of all RSV hospitalisations during the first two years of life 58 children (64 admissions) were born before completed 37 weeks of gestation with a median gestational age of 30 weeks (range 24–36 weeks). A significant male predominance of 68% was also observed in this group (P = 0.006). Median age on admission, after postnatal age, was 8 months (range 0–23 months). When corrected for prematurity (corrected age), median age on admission was 5.4 months (range -2.5–21.3 months). Also, a median length of hospitalisation of 8 days (range 2–27 days) was observed (table 1). Two of the preterm children (3.4%) needed mechanical ventilation and two were treated with inhaled nebulized ribavirin [10]. No preterm children in this study died from RSV bronchiolitis.

Seven children (eight admissions) diagnosed with trisomy 21 were hospitalised for RSV bronchiolitis during this follow-up study. Four of them also had a CHD. Two children needed mechanical ventilation during hospitalisation and one of them subsequently died.

A median age on admission of 9.0 months (range 1–20 months) and a median stay of 7.5 days (range 2–34 days) were recorded for children in this particular risk group (table 1).

A total of 20 children had a CHD, four combined with prematurity, four combined with trisomy 21, and 12 with no other additional risk-factor for severe disease. In the group of children with only CHD as risk factor median age on admission was 7.0 months (range 0–23 months) and median stay of 6.0 days (range 2–14 days) was observed (table 1). One child needed mechanical ventilation and subsequently died.

### Hospitalisation incidences

Overall hospitalisation incidences were calculated from the known number by official Norwegian statistics of 58.179 children under two years of age living in the service area for the whole study period as well as for each year as given in table 2. As shown, the overall incidences for the whole study population was 21.7 admissions per 1.000 children under one year of age, 6.8 admissions per 1.000 children 1–2 years of age and 14.1 admissions per 1.000 children under two years of age, with some year to year variation (table 2).

**Table 2:** Mean annual hospitalisation incidences per 1.000 children by age and risk-groups in children under two years of age with RSV bronchiolitis in Akershus, Norway, 1993–2000

	Children <1 year			Children 1–2 years			Children 0–2 years		
Year^a^	Pop^d^	Adm	Inc	Pop^d^	Adm	Inc	Pop^d^	Adm	Inc

All 93–94	4.027	70	17.4	4.195	16	3.8	8.222	86	10.5
All 94–95	3.999	53	13.3	4.192	25	6.0	8.191	78	9.5
All 95–96	3.994	112	28.0	4.132	30	7.3	8.126	142	17.5
All 96–97	4.055	80	19.7	4.143	22	5.3	8.198	102	12.4
All 97–98	4.154	115	27.7	4.242	57	13.4	8.396	172	20.5
All 98–99	4.178	70	16.8	4.350	15	3.4	8.528	85	10.0
All 99–00	4.127	120	29.1	4.391	37	8.4	8.518	157	18.4
All 93–2000	28.534	620	21.7	29.645	202	6.8	58.179	822	14.1
Premature^b ^93–2000	1.999^c^	47	23.5	1.955^c^	17	8.7	3.954^c^	64	16.2
Trisomy 21 93–2000							52	8	153.8

^a^Study period February 5th, 1993 to January 31th, 2000

^b^Born at <37 weeks of gestational age

^c^Number of children born at <37 weeks of gestation and corrected for a mean infant mortality rate of 2.4% during the neonatal period

^d^Background population for selected risk groups

Data from the Norwegian Birth Registry identified that 1.999 infants born in the service area before completed 37 weeks of gestation were alive and below 1 year of age, and 1.955 infants were alive and between 1–2 years of age during the study period. As shown, the corresponding hospitalisation incidences for preterm children were 23.5, 8.7 and 16.2 per 1.000 children under one year of age, 1–2 years of age and <2 years of age respectively (table 2).

From the hospital records 52 children under two years of age were diagnosed with trisomy 21 during the follow-up period. Among these seven children (eight admissions) were hospitalised for RSV bronchiolitis, giving a hospitalisation incidence under two years of 153.8 per 1.000 children.

## Discussion

In this retrospective study, as in many other comparable epidemiological studies, we found that RSV bronchiolitis appears as an annual winter epidemic with relatively high hospitalisation incidences, predominance of boys, young age, short length of stay, few complications and low mortality [1]. The rather long study period makes the results less vulnerable for yearly variations in magnitude of the annual epidemic and in virulence of the microbe. By using NPA for microbiological diagnosis in all children with symptoms of a lower respiratory tract infection like tachypnoe, wheezing or apnoic spells we believe that the great majority of cases are included. However, the enzyme-linked immunosorbent assay used in the present study could give both false positive and false negative results [9,11].

Several international studies have shown an increased incidence of RSV-related hospitalisations over the last two decades. A Norwegian study for the period 1972–78 showed a mean hospitalisation incidence of 9.5 per 1.000 children for children under one year of age compared to our much higher incidence of 21.7 per 1.000 children for the period 1993–2000 in the same age group [12]. Also, for children 1–2 years old the incidence in our study was much higher. A large study from USA for the period 1980–96 showed an increased incidence rising from 12.9 per 1.000 children in 1980 to 31.2 per 1.000 children in 1996 for children under one year of age [13]. However, comparing historical data is difficult, and for the period 1993–2000 we observed no increase in the mean annual hospitalisation incidences of RSV bronchiolitis. Factors such as increased microbial virulence, increased day-care attendance, improved microbiological diagnosis and more precise ICD-coding might all be important factors to explain these historical differences. Number of hospitalisations are also related to the severity of the clinical symptoms. Thus, changes in hospital admission policies over time as well as changes in availability of hospital services might also influence hospitalisation incidences. A recent study from USA for the period 1997–99 showed that RSV bronchiolitis was the leading cause of hospital admissions of infants younger than one year of age with an associated hospitalisation incidence of 25.2 per 1.000 infants. This is in accordance with our findings [14].

Our study showed that only 1.2% of the children in the study population needed mechanical ventilation. This result is lower than in many other studies [15]. On the other hand, when children were in need of mechanical ventilation, the risk of severe outcome was in our study very high, with two out of nine mechanically ventilated children subsequently dying. A Danish study showed that only 0.6% of their children needed mechanical ventilation, however, 20% of all children with severe respiratory failure were given ventilatory support with nasal continuous positive airway pressure (N-CPAP) and some infants possibly avoided mechanical ventilation for that reason [16]. More controlled studies on different treatment strategies are very much needed, especially when considering how to avoid respiratory failure and mechanical ventilation [17,18].

Another observation in our study was that among the mechanically ventilated children 44% had no underlying risk-factors for severe disease. Further research into why some otherwise healthy children have a severe course of RSV bronchiolitis is therefore very important, not least when trying to define risk groups even better [19]. Research for a better understanding of the host immune response to RSV bronchiolitis the later years might be one important piece in this puzzle [20-22].

For the population at large our study showed, as in other studies, a low mortality. A total of two children (0.3%) died, one had trisomy 21 with an atrioventricular septum defect (AVSD) and the other had an underlying CHD.

The number of high-risk children in our study is relatively small and the results should be interpreted with caution. The mean hospitalisation incidences for preterm children were lower than in many other studies, even though 20% of the preterm children suffered from CLD as well and had a low median weeks of gestational age (wGA) of 30. One possible explanation is that the population data for this particular risk group was not corrected for migration out of our service area and mortality after the neonatal period. However, it is unlikely that such corrections would significantly influence the results. Another explanation for the low incidence among preterm infants might be that most of them are hospitalised up to almost 40 wGA and are discharged only when the child has reached a weight of more than 2.000 g and have normal oral feedings. This means that they are more or less completely protected from RSV for a long period after birth. Our routines with focus on good parental information on how to protect the newborn infant from RSV after discharge from hospital might also be important. Median length of hospitalisation for preterm children was in our study significantly longer when compared to the low-risk children and is in accordance with results from many other larger studies.

Children with CHD are a wellknown risk-group for severe RSV disease. Children with trisomy 21 with or without a concomitant CHD should also be considered a high-risk group. Our study showed a mean hospitalisation incidence per 1.000 children under two years of age with trisomy 21 more than 11 times the whole study population. Even more important, out of a total of seven children with trisomy 21, two needed mechanical ventilation and one child died. We would, however, recommend a larger study to meet these observations for children with trisomy 21.

One of the findings in our study was the large proportion of otherwise healthy children needing mechanical ventilation. Our numbers are small, but when new policies to prevent severe RSV bronchiolitis are discussed this aspect should also be considered. The research for improved identification of risk-groups and development of an effective vaccine for immunization should therefore be given high priority.

## Conclusions

While the overall hospitalisation incidences and outcome of RSV bronchiolitis were in agreement with other studies, hospitalisation incidences for preterm children were lower than in many other studies. Age on admission for preterm children, when corrected for prematurity, was comparable to low-risk children. Length of hospitalisation and morbidity was high in both preterm children, children with a congenital heart disease and in children with trisomy 21, the last group being at particular high risk for severe disease.

## Competing interests

The author(s) declare that they have no competing interests.

## Authors' contributions

Our individual contributions to the study have been as following: HOF had primary responsibility for protocol development, outcome assessment, data acquisition and analysis and writing of the manuscript. TF participated in the development of the protocol and analytic framework of the study, and contributed to the writing of the manuscript. DB participated in the data analyses and the writing of the manuscript. All authors read and approved the final manuscript.

## Pre-publication history

The pre-publication history for this paper can be accessed here:


